# Transitioning from donor aid for health: perspectives of national stakeholders in Ghana

**DOI:** 10.1136/bmjgh-2020-003896

**Published:** 2021-01-13

**Authors:** Wenhui Mao, Kaci Kennedy McDade, Hanna E Huffstetler, Joseph Dodoo, Daniel Nana Yaw Abankwah, Nathaniel Coleman, Judy Riviere, Jiaqi Zhang, Justice Nonvignon, Ipchita Bharali, Shashika Bandara, Osondu Ogbuoji, Gavin Yamey

**Affiliations:** 1 Center for Policy Impact in Global Health, Duke Global Health Institute, Duke University, Durham, North Carolina, USA; 2 Policy Planning Monitoring and Evaluation Directorate, Policy Coordination Unit, Ghana Ministry of Health, Accra, Greater Accra, Ghana; 3 School of Public Health and Family Medicine, University of Cape Town, Rondebosch, Western Cape, South Africa; 4 Department of Social and Behavioral Sciences, University of Ghana School of Public Health, Accra, Greater Accra, Ghana; 5 University of Ghana School of Public Health, Accra, Greater Accra, Ghana

**Keywords:** health policy, qualitative study

## Abstract

**Background:**

Ghana’s shift from low-income to middle-income status will make it ineligible to receive concessional aid in the future. While transition may be a reflection of positive changes in a country, such as economic development or health progress, a loss of support from donor agencies could have negative impacts on health system performance and population health. We aimed to identify key challenges and opportunities that Ghana will face in dealing with aid transition, specifically from the point of view of country-level stakeholders.

**Methods:**

We conducted key informant interviews with 18 stakeholders from the government, civil society organisations and donor agencies in Ghana using a semistructured interview guide. We performed directed content analysis of the interview transcripts to identify key themes related to anticipated challenges and opportunities that might result from donor transitions.

**Results:**

Overall, stakeholders identified challenges more frequently than opportunities. All stakeholders interviewed believe that Ghana will face substantial challenges due to donor transitions. Challenges include difficulty filling financial gaps left by donors, the shifting of national priorities away from the health sector, lack of human resources for health, interrupted care for beneficiaries of donor-funded health programmes, neglect of vulnerable populations and loss of the accountability mechanisms that are linked with donor financing. However, stakeholders also identified key opportunities that transitions might present, including efficiency gains, increased self-determination and self-sufficiency, enhanced capacity to leverage domestic resources and improved revenue mobilisation.

**Conclusion:**

Stakeholders in Ghana believe transitioning away from aid for health presents both challenges and opportunities. The challenges could be addressed by conducting a transition readiness assessment, identifying health sector priorities, developing a transition plan with a budget to continue critical health programmes and mobilising greater political commitment to health. The loss of aid could be turned into an opportunity to integrate vertical programmes into a more comprehensive health system.

**Key questions**

**What is already known?**

- When not properly managed, donor transitions can affect the different components of the health system, lead to disruptions in service delivery and increase the risk of disease resurgence.
- Upcoming cohorts of countries that will graduate from multilateral donor assistance in the coming years, including Ghana, have less capacity than previous graduates to manage donor transition.
- Additionally, the transition process for upcoming graduates could become even more challenging since these countries are also experiencing substantial demographic changes, an epidemiological transition and a shifting domestic financing landscape.

**What are the new findings?**

- Overall, stakeholders identified challenges more frequently than opportunities.
- Challenges include difficulty filling financial gaps left by donors, the shifting of national priorities away from the health sector, lack of human resources for health, interrupted care for beneficiaries of donor-funded health programmes, neglect of vulnerable populations and loss of the accountability mechanisms that are linked with donor financing.
- Stakeholders also identified key opportunities that transitions might present, including efficiency gains, increased self-determination and self-sufficiency, enhanced capacity to leverage domestic resources and improved revenue mobilisation.

**What do the new findings imply?**

- The challenges could be addressed by conducting a transition readiness assessment, identifying health sector priorities, developing a transition plan and mobilising greater political commitment to health.
- The transition from aid could be turned into an opportunity to integrate vertical programmes into a more comprehensive health system.

## Introduction

Ghana has undergone rapid economic development, and in 2010, it moved from being a low-income country (LIC) to a lower middle-income country (LMIC). Between 2000 and 2018, its gross national income (GNI) per capita multiplied more than six times.^1^ However, this impressive economic development has not been entirely matched by improvements in Ghana’s health financing or population health outcomes.^2^ Current health expenditure accounted for 3.3% of gross domestic product (GDP) in 2017 and 40.3% of health expenditure came from out-of-pocket payments.^3^ In health outcomes, Ghana achieved the Millennium Development Goal (MDG) 1 target of halving the poverty rate from 1990 to 2015, but was unable to meet the MDG 5 target of reducing the maternal mortality ratio by three-quarters over the same time period. HIV/AIDS remains a major cause of morbidity and mortality. Ischaemic heart disease rose from being sixth on the list of top 10 causes of death in 2007 to fourth on the list by 2017.^4 5^ With an ageing population,^6^ Ghana is faced with a ‘double burden’ of disease from both infectious diseases and non-communicable diseases (NCDs) and an unfinished MDG agenda of high child and maternal mortality.

In 2019, Ghanaian President Nana Akufo-Addo announced his government’s policy agenda for moving Ghana ‘Beyond Aid’.^7^ The intention of this policy is to further the discussion about moving away from development aid for critical functions, such as the health and education system, towards self-reliant sustainable financing. A decreasing reliance on foreign aid, whether driven by a donor or a recipient country, can be referred to as transition from aid. In particular, in this paper, we refer to transition as a change in an external funder’s policy, financing level or programming with the intention of giving more responsibility to a country to sustain the health gains from external funds in its preparation for an era beyond aid.^8^

Realising a self-reliant health system in Ghana will be a challenge.^9^ Although Ghana primarily funds its health system through domestic government funds and out-of-pocket payments, in 2017, development partners funded 19% of all current health expenditures. By 2022, the external share is expected to fall to only 1% of current health expenditures.^10^ However, even before the ‘Beyond Aid’ agenda, Ghana struggled to meet its co-financing commitments for Gavi, the Vaccine Alliance (Gavi),^11^ one of several major external health funders in the country (more details on Gavi and the role of other major external financers of health are in online supplemental appendix I). To fill this gap, massive domestic resources will need to be mobilised and careful transition planning will need to take place to ensure development gains that have been funded by external players are maintained. The National Health Insurance Scheme could be one of the channels that could be further used to increase domestic spending on health in Ghana; however, there are emerging concerns about the sustainability of the scheme.^12^

10.1136/bmjgh-2020-003896.supp1 Supplementary data

When not properly managed, donor transitions can affect the different components of the health system in unique and varied ways. Some countries may lack the human resources or technical capacity needed to continue activities formerly led by donors, such as medical product procurement or programme management.^13 14^ Parallel systems for service delivery (eg, delivering HIV or maternity services) may have been established by donors without sufficient local buy-in, and therefore, donor exits may leave a vacuum for particular populations. Each of these challenges can lead to disruptions in service delivery, which can increase the risk of disease resurgence.^15^ An analysis by Yamey *et al* found that the upcoming cohort of countries that will graduate from multilateral donor assistance in the coming years, including Ghana, have less capacity than previous graduates to manage the donor transition.^16^ They found that “the upcoming cohort seems to have, on average, lower per capita income, greater indebtedness, weaker capacity to efficiently use public resources, more limited and less effective health systems, weaker governance and public institutions, and greater inequality.” Additionally, the transition process for upcoming graduates could become even more challenging since these countries are also experiencing substantial demographic changes, an epidemiological transition and a shifting domestic financing landscape.^17^

Research on transitions from donor assistance for health have primarily examined the perspective of bilateral and multilateral donors and certain disease areas. The role of recipient countries in transition planning and the concerns of recipient countries may have been under-represented.^18^ To identify the challenges and opportunities in transition from health aid from a recipient country’s perspective, we conducted a qualitative study of stakeholders in Ghana to understand their perspectives as the country navigates the transition process.

## Methods

### Study design

We used a cross-sectional qualitative design. We chose a qualitative approach because it helps ‘to answer questions about experience, meaning and perspective, most often from the standpoint of the participant’.^19^ We used this design to explore how in-country stakeholders understood the transition process and what they perceived as the prospects for and challenges of donor transitions in the health sector.

### Setting

We selected Ghana for this qualitative study of stakeholders’ views on transition, since Ghana is a transitioning lower middle-income country that could experience unintended effects if donors and the government fail to manage transition carefully. Ghana has been receiving health aid from many different donors since the 1990s.^20^ Due to its recent economic growth, Ghana is one of around two dozen countries that in the coming years are likely to face simultaneous donor transitions (ie, the loss of assistance from multiple donors at once).^21^ In addition, Ghana is undergoing an epidemiological transition (a shift in the burden of disease from infectious diseases to NCDs), a demographic transition (eg, ageing of the population) and a number of obstacles in achieving universal health coverage.^22^ These factors typify the challenges that LMICs face as they lose development assistance for health (DAH). In addition, given that Ghana has committed to achieving universal health coverage (UHC),^23^ it is also important to explore if there are strategies available to Ghana to mitigate the risk of transition while progressing towards UHC.

### Sampling technique

We used a purposive sampling technique to select stakeholders in government (including Parliament, Ministry of Financing, Ministry of Health and Ministry of Planning), civil society organisations and donor agencies in Ghana. We selected participants who had knowledge of and/or experience with donor funding, or were familiar with ongoing donor transitions in Ghana’s health sector. Based on the in-depth knowledge of two Ghanaian researchers in the team (JNOD and JN), we developed an initial list of potential key informants (KIs) who were selected to represent different stakeholder groups. Additional respondents were identified through snowballing. We requested the participation of interviewees either in person or via telephone/email using a standard script. Follow-up contact was attempted for participants who did not initially respond.

We conducted interviews until saturation was reached. Our final sample included 18 KIs: 10 represented government institutions (8 from the national level and 2 from the regional level), 7 represented civil society organisations and 1 worked for a donor agency at the country level (online supplemental appendix II).

### Data collection

We conducted semistructured interviews with 18 KIs. The interviews were conducted in person by two Ghanaian researchers in the team (DNYA and NC) with experience in conducting qualitative interviews.

We used a semistructured interview guide that was piloted for feasibility and acceptability by members of the research team based in Ghana (online supplemental appendix III). Questions focused on the biggest current or potential challenges and opportunities related to Ghana’s donor transition in the health sector. Interviewers asked probing questions about the challenges and opportunities presented by transition out of DAH. Probing questions were guided by the WHO framework for health systems (particularly, the six health systems building blocks: service delivery, human resources, essential medicines, health financing, health information systems and governance).^24^ Prior to the start of the interview, all interviewees provided consent to be audio-recorded. All interviews were conducted in English and transcribed. They were conducted in-person at the offices of interviewees, and lasted an average of 47 min (range 17–80 min).

### Data analysis

We performed directed content analysis (also called deductive content analysis) of the interview transcripts to characterise KIs’ views on the challenges and opportunities related to donor transitions.^25^ With a directed approach, analysis starts with a theory or relevant research findings as guidance for initial codes. Given that we used the WHO framework to ask probing questions related to the challenges and opportunities of donor transitions, a deductive approach was most appropriate to address our specific research question focused on the perspectives of stakeholders in Ghana.

Once interviews were transcribed by DNYA and NC, two members of the study team read through all the transcripts several times to better understand the nature of the data as a whole and to develop a codebook.^12^ The codebook contained structural codes, based on the interview guide questions, and thematic codes, based on emerging themes from the transcripts. Within these primary codes, subthemes were identified from each transcript. Next, if similar topics were frequently mentioned by interviewees, we integrated the thematic codes. To ensure the robustness of the codebook, team members piloted the codebook on three interview transcripts and discussed any discrepancies in coding. After the codebook was finalised, all interview transcripts were independently coded by two team members, who met after coding each transcript to assess inter-coder reliability. Coding discrepancies were resolved by consensus and if the two coders did not agree, they consulted with a third to make the final decision. Results presented are based on the most prevalent codes. However, we include details and nuance within each of the codes to present the variety of interpretations and views stakeholders expressed. NVivo V.12 software was used to organise and index codes and to complete the coding process.

### Ethical considerations

Written informed consent was obtained from all participants prior to the interview. If the participant consented to being audio-recorded, interviews were audio-recorded and deidentified. To protect KIs’ confidentiality, we have included no identifying information about them in this paper.

## Results

Overall, KIs held mixed views on the impacts of Ghana’s transition from health aid. Some participants—largely policy makers—felt positively about transitioning from aid, framing it as an opportunity for Ghana. Other participants—largely implementation partners—expressed negative sentiments about transition. A few interviewees even described feeling ‘forced’ into transition, meaning a transition would happen regardless of the readiness of the country.

There was a general consensus among KIs from Ghana that transition should involve all kinds of stakeholders, including individuals from the different ministries (health, financing and planning), donor organisations, implementation partners at district and facility levels, as well as the private sector. Most participants expressed that the transition should be a collaborative process that involves coordination between the donor and the country, but donors and countries have separate responsibilities that they should both uphold.

In-country stakeholders identified many challenges and opportunities associated with donor transitions in Ghana. Overall, stakeholders identified challenges more frequently than opportunities.

### Challenges

All KIs believed Ghana will face many critical challenges due to donor transitions. A variety of challenges were highlighted, and primarily fell under five major themes: (1) financial gaps left by donors; (2) shifting national priorities; (3) loss of the technical capacity that accompanies aid; (4) service delivery interruptions, particularly for neglected/vulnerable populations; and (5) reductions in monitoring, evaluation and accountability.

#### Financial gaps left by donors

All stakeholders pointed to funding gaps as one of the biggest challenges that the country could face during donor transitions. In particular, all interviewees expressed concerns that the transition would lead to gaps in funding for areas that are currently mostly funded by donors, such as vertical HIV control programmes, health commodities/supplies (eg, vaccines), health workforce training/capacity building, infrastructure, technology and logistics.

We used to have donors building health facilities for us … so when the donors leave, we face serious challenges running the health sector. All the support withdraw[n] now [is] having serious impacts on service delivery.—KI 01

Most stakeholders believed that when donors eventually leave Ghana, domestic resources alone will not be sufficient to sustain progress. There was additional concern among KIs that this inadequate domestic financing may be compounded by Ghana’s limited financial management capacity, such as revenue generation.

So, I will say that the state tries to cover up all the gaps left by the donors, but because of the reality of not getting all the budget amount that we do, we are unable to do it [cover all the financial gaps left by donors] fully but there are efforts to try and cover it [the financial gaps] up, but it is not 100% achieved.—KI 09

Half of the KIs expressed concerns that since donors would no longer pay for certain supplies after transition, the cost will have to be borne by the government and/or service users via out-of-pocket payments. If this occurs, KIs fear it will affect both access to, and quality of, health services.

#### Shifting national priorities

Most stakeholders felt that it is challenging to manage transition in a changing political environment. Ghana has an election every 4 years and health policies have become vote-winning strategies. When a new party or minister comes into power, KIs feared that the ‘old programme’ would not survive under the new leadership. Without continuous donor-funded health programmes, even if some donor-funded programmes would be supported by the current government immediately after donor transition, there is no guarantee that those programmes will keep receiving the same level of support from the next government. In addition, KIs said that when donors leave, certain health issues no longer receive the same level of attention.

Probably the priorities of the government in power would bring challenges to service delivery, for instance if they think infrastructure development is more important than health, then of course health care would suffer [receive less attention/resources].—KI 16

#### Loss of technical capacity

Almost three-quarters of stakeholders expressed concern that donor exits could lead to loss of technical expertise and of funding for capacity building. While some interviewees felt that the government of Ghana would fill in the capacity gap left by donors, stakeholders still perceived a risk that many health professionals will not remain in Ghana’s health system if there is a gap in time between donor transition and government support. Training, often funded by donors, is likely to be discontinued, and stakeholders expressed that this would, in turn, negatively affect the capacity of the health system.

[Technical capacity] will be affected because the government might not be able to provide training needed to equip the human resources needs of the service. As donors contribute to these trainings, their [the donors’] transition will definitely affect the human and technical capacity of the nation.—KI 14

#### Interruption in healthcare and neglected vulnerable populations

Half of the interviewees flagged concerns about interruptions in health services after transition, which could, in turn, affect health outcomes. Specifically, interviewees were concerned that public health services, including maternal and child health, HIV care, malaria control, vaccines, and outreach services, would be more affected than other health services.

For EPI [the Expanded Program on Immunization], it [transition] will impact greatly because we know that every child is supposed to be vaccinated and when there is a cut in supply of these vaccines, definitely there will be a threat in our immunization cycle and that will affect the nation as a whole.— KI 17

A less frequently raised concern was that transition could lead to reduced access to and use of health services, which may reverse prior achievements in health. For example, two stakeholders believed that herd immunity may be lost if there is a subsequent reduction in the coverage of vaccine programmes after Gavi exits.

Furthermore, some interviewees raised concerns that vulnerable populations may suffer the most from donor transition.

The public facilities provide services to these vulnerable populations so when we are not having the funding to provide the healthcare they [the vulnerable populations] will be in trouble.—KI 13

#### Reductions in monitoring, evaluation and accountability

Nearly half of stakeholders felt that donor transitions could reduce support for monitoring and evaluation (M&E) activities. They believe the M&E and even auditing practices that are often integrated into donor programmes are helpful in improving quality of health services. Stakeholders felt that the Government of Ghana is unlikely to have the capacity or resources to provide similar levels of M&E after transition. They further anticipate that this loss of M&E support would reduce the accountability and efficiency of the health delivery system. Without consistent M&E, stakeholders believe it would be difficult to identify programme gaps, make strategic plans and avoid corruption.

Now, [because of donor exits], our M&E is gone. I don’t think for health they even have an M&E. It is when you monitor that you see where the gaps are. So, we need to monitor to see where the challenges are. We need to make sure that our M&E is more robust because that would show that the resources that we have, meant for a particular purpose, has actually met it.—KI 11

### Opportunities

In-country stakeholders identified a number of opportunities presented by donor transitions. Overall, KIs commonly expressed sentiments of increased efficiency (ie, doing more with less) and enhanced feelings of self-determination (ie, control over priorities). Stakeholders also frequently flagged key opportunities to better leverage existing resources and enhance revenue mobilisation for the health sector.

#### Efficiency gains

Two-thirds of the interviewees highlighted that donor transitions would enable Ghana to become more efficient with its resources. Efficiency was typically referred to in the context of maximising resources and was primarily expressed in three ways: improved prioritisation, reduction of wasteful spending and more creative problem solving.

##### Improved prioritisation to achieve greater alignment between needs and spending

Two-fifths of stakeholders expressed the view that donor financing has historically targeted certain programmatic areas (eg, HIV/AIDS), neglecting other potentially more important domestic priorities. According to these interviewees, relying on domestic resources and partners, alongside domestically driven priorities, may lead to more alignment between perceived needs and spending during and following transition.

It is our country and if someone is helping and the person decide[s] to leave you then you have to change your game plan. The opportunities are that you have to re-prioritize, look at issues of efficiency, the way we operate and all that are in-house opportunities. We need to evaluate the program and I don’t think all the programs that come with money to us are really necessary.—KI 05

##### Reduction of wasteful spending

Nearly half of the interviewees argued that when there is an abundance of donor-provided funds, there is more likely to be waste or leakages within the health sector. These interviewees believe that turning off this influx of external resources is a way to reduce spending that is not in alignment with needs. ‘Waste’ was also referred to as the misuse of services when a patient did not have sufficient need, as well as the duplication of programmes within the health system due to parallel donor systems.

I will say that if today we are out of donor [support] I will be happy. The reasons are that people will not get the chance to misuse the funds.—KI 05

##### Creative problem solving

Doing more with less was viewed by a third of interviewees as an opportunity during and following transition since it may lead to more creative solutions to problems. Without an abundance of resources, these stakeholders felt that they would be empowered to find new ways to do more with less, such as through programme integration.

I am not saying people should be poor but when there [is] no money it allows people to think. Because, sometimes in the health sector, when money runs out in a program then people begin to suggest that we should integrate the programs and work together.—KI 06

#### Increased self-determination and self-sufficiency

Two-thirds of stakeholders emphasised the greater sense of independence and self-determination that donor exits would bring. Self-determination was described as the ability to self-select the appropriate course of action; self-sufficiency, while closely linked to self-determination, was viewed as the ability to rely on one’s own skills or resources.

##### Self-determination

Although external support is appreciated, nearly half of the interviewees expressed readiness to focus on areas in accordance with domestic policies and priorities following transition. Donor funds, they argued, can often be limiting, conditional or otherwise restrictive. Spending and programming in accordance with domestic priorities may bring a greater sense of self-determination.

I think as a country we would have the opportunity to decide the future for ourselves and focus on what we really want to do and achieve … so you could see clearly that someone was influencing that activity but now that the money is coming from us we can now actually decide what we want to do and that can influence some of the policies we want to take.—KI 16

##### Self-sufficiency

Interviewees also expressed readiness to rely on their own domestic capacity. Donors have funded capacity building and development in the past, but now some stakeholders mentioned they have the opportunity to put their skills into practice.

Now I think the country can start looking inward at what it can produce to support itself rather than rely on donor support to bring in what we think we need or what they produce outside.—KI 16

#### Enhanced capacity to leverage existing domestic resources

Two-fifths of stakeholders shared a strong sense that Ghana already has the resources it needs to support its health system (eg, financial, human and so on), but perhaps has not tapped into them sufficiently due to the role donors have played. If donors exit, these stakeholders believe that there is an abundance of resources that could be leveraged. In addition to financial resources, stakeholders mentioned they could forge new domestic partnerships or tap into Ghana’s favourable policy environment or even new oil revenues.

… as a result of the donors moving out we have been forced to approach other stakeholders and we were surprised that they were willing to help. They were wondering why we never went to them in the first place but we were also getting it for free so we never bothered. So it will give us the opportunity to interact with other stakeholders which are internal and I think that will allow us to redefine our client care to suit our people.—KI 16

#### Improved revenue mobilisation

About half of interviewees said that revenue generation may improve after donor transitions. Donors, they said, may have inadvertently disrupted domestic healthcare markets and demand by providing products and services for free. However, after donor exits, the private sector and hospitals may have an opportunity to profit from such products and services.

We should … look inwards and see how we can help to mobilize local resources to support when they leave. There are companies out there … how do we get to them with attractive packages and programmes so that they can help to support?—KI 05

## Discussion

Overall, interviewees from Ghana identified substantial challenges presented by donor transition, including major financial gaps that could impact Ghana’s health system. Shifting national priorities and the loss of accountability that donors tend to impose on donor-funded health programmes could make it hard for Ghana to fill the gaps left by transition. The lack of continuity of government-driven policies and programmes is a major challenge that extends beyond health sector. Access to high quality and continuous care may be negatively affected, especially for programmes currently receiving heavy donor subsidies as well as programmes for vulnerable populations. Reductions in capacity building opportunities and declines in funding for human resources for health are also concerning issues during donor transition.

Despite these potential challenges, interviewees highlighted that transition presents clear opportunities. Ghana’s health system may see enhanced efficiency due to better prioritisation in the absence of donor influence, reductions in waste and creative problem solving for ways to maximise domestic resources. An increased sense of self-determination may help guide the health system according to domestic, rather than external priorities, and lead to leveraging of existing, but underused resources. Additional sources of revenue for the health system could be mobilised when there is more competition (ie, when free services/commodities from donors are eliminated) and when there is fair pricing.

Our study is timely given the government of Ghana’s recent commitment to achieve ‘Ghana Beyond Aid’.^6^ Although donors may unilaterally choose to transition out of a recipient country, Ghana’s commitment to operate beyond aid establishes a framework through which Ghana can assume ownership and drive the transition process. The Ghana Beyond Aid commitment is country-wide and not specific to the health sector. Therefore, our findings may help support the application of the Ghana Beyond Aid agenda within the health sector by identifying key vulnerabilities and highlighting potential opportunities that may help mitigate some of the challenges.

The government could take four key steps to mitigate challenges and harness opportunities presented by transition in the health sector:

1. Conduct a health sector transition readiness assessment. The stakeholders that were interviewed for this study clearly know the common challenges and opportunities Ghana could face in a transition. However, a formal evaluation of key areas of vulnerability could be conducted to identify how potential pitfalls can be mitigated or avoided. Several existing transition readiness assessment frameworks could be drawn upon, such as the Programmatic Mapping Readiness Assessment for Use with Key Populations, Guidance for Analysis of Country Readiness for Global Fund Transition and the Transition Preparedness Assessment Framework.^26–28^ In 2018, the UK Department for International Development (now merged into the Foreign, Commonwealth & Development Office) funded a study on sustainable funding for key disease areas funded by donors, which could be a helpful input into a more comprehensive health sector transition assessment. The World Bank has also conducted a comprehensive assessment of Ghana’s health system, which could also be incorporated into a health transition readiness assessment.^29^
2. Identify health sector priorities. Donors may have inadvertently influenced the prioritisation process by encouraging greater priority to certain areas over others or causing a shift in domestic resources to areas neglected by donors. Transition provides a unique opportunity to revisit Ghana’s health system and priorities by evaluating the health needs of the population and identifying areas that may require greater investment given the reduction or absence of donor funds. Priority setting enables the system to adapt to the changing financing landscape and can guide strategic planning for the health sector beyond transition. This is particularly salient for Ghana given the simultaneous shifts in its demographic and disease burden, which put new strains on the health system.^19^
3. Develop a health sector transition plan. The health sector needs to outline a clear approach to addressing the challenges and vulnerabilities identified in the readiness assessment, which should be guided by Ghana’s updated health sector priorities with budgeting to continue critical health programmes. Strategies to address the major challenges identified in this study, including funding and capacity gaps, interrupted health services and loss of external accountability, should be considered. Additionally, with a transition plan in hand, activities that would promote a smooth transition, such as close collaboration with development partners in sharing information, undertaking capacity building, and monitoring and evaluation, are more likely to be supported during the transition process. Several transition plans have been developed for previous health-centric transitions that could be drawn upon.^17–19^
4. Advocate for greater government commitment to the health sector. Case studies from other countries have shown that when governments are not willing to fill the technical and financial gaps left by donor exits, disease resurgence is possible.^9^ For example, after the Global Fund exited Romania, the government failed to maintain HIV prevention services for people who inject drugs and the HIV prevalence among this group rose sharply.^30^ Given stakeholders’ concerns for shifting priorities and loss of programming for vulnerable populations, the government should intensify its commitment to support the health sector during and after transition to avoid such outcomes. The government should revise the budget allocation ceilings in the medium-term given the sector’s anticipated resource reductions.

Although many disease-specific programmes, such as HIV or vaccination programmes, are expected to lose major sources of financing when donors leave, it is possible to convert the loss of DAH into an opportunity to integrate these vertical programmes into a more comprehensive health system. This integration would require massive resource mobilisation, strong political commitment and continuous efforts from the government. Sparkes and colleagues at the WHO have developed a system-wide analytic approach to drive such integration of vertical programmes and improve efficiency, and it would be valuable for Ghana to undertake such an analysis.^31^ Conducting such an analysis, say Sparkes and colleagues, can provide “the foundation to identify potential opportunities and options to get more or better coverage from available resources through reconfiguration, which may include new investment in underlying cross-cutting aspects as relevant.”

Based on the concerns raised by stakeholders in Ghana, donors, on the other hand, could mitigate the challenges associated with transition by (1) communicating with countries about transition plans as early as possible; (2) engaging in transition planning with in-country stakeholders; and (3) supporting activities to strengthen the overall health system.

To the best of our knowledge, this is the first study to present health aid transition challenges and opportunities from the country perspective. It is critical to understand the views of national policy makers, not just donors, since it is national stakeholders who must maintain or even intensify health programme activities after donors leave. We interviewed a wide range of informants across different sectors, and we reached theoretical saturation, so it is likely that we captured most of the key views about transition among country policymakers.

Nevertheless, as with any qualitative study of this kind, our study also had a number of limitations—we highlight three in particular. First, this study examined the experiences of stakeholders in Ghana alone, and the findings may not be generalisable to every country facing donor transition. Second, while our study laid out the challenges and opportunities associated with transition, we have not analysed information from interviewees for strategies that could best lead to a smooth transition process. Additional research is needed to explore and evaluate such strategies. Finally, we only interviewed one stakeholder from a donor agency based in-country. Therefore, we likely under-represent the perspective of this group.

## Conclusion

Stakeholders in Ghana believe transitioning away from health aid presents both challenges and opportunities. Conducting a transition readiness assessment, identifying health sector priorities, developing a transition plan with a budget to continue critical health programmes and maintaining political commitment to health could address the challenges. The loss of DAH could be transformed into an opportunity to integrate vertical programmes into a more comprehensive health system.
