# Supplementary material for: Transitioning from donor aid for health: perspectives of national stakeholders in Ghana
Source: BMJ Glob Health. 2021 Jan 13;6(1):e003896. doi: 10.1136/bmjgh-2020-003896 (PMC7812084; doi:10.1136/bmjgh-2020-003896)
Supplement: Supplementary data [file bmjgh-2020-003896supp001.pdf]

Appendix I The role of external aid donors in Ghana's health system

Ghana is expected to decrease its reliance on health aid from nearly 19% of current health expenditures to only 1% by 2021 [8]. However, several factors may complicate this transition to a health system that operates beyond aid.

Ghana has a very concentrated donor environment. Donor concentration is defined as four or fewer donors making up more than 50% of aid. In 2018, two donors made up more than 50% of health aid in Ghana, demonstrating a very concentrated environment. Overall, seven donors account for the vast majority (95%) of the total health aid portfolio in Ghana: the United States (US) (28%), the Global Fund to Fight AIDS, Tuberculosis and Malaria (the Global Fund) (27%), Gavi (14%), Canada (9%), Japan (8%), the World Bank International Development Association (IDA) (4%), and the United Kingdom (UK) (4%) [1]. These same donors have consistently made up more than 75% of all health aid since 2002.

Only some of Ghana's major donors have a clear transition policy and approach. Ghana is expected to enter into Gavi's accelerated transition phase in 2021, a phase that tends to last around five years. This means Ghana could be expected to fully self-finance its vaccines program by 2026. Although Ghana has increased its share of financing for routine immunization from 16% in 2013 to 25% in 2017, Ghana has twice defaulted on its co-financing commitments to Gavi [**Error! Bookmark not defined.**,2].

Overall, IDA funding has declined as a major source of health funding in Ghana, from over 40% of total health ODA from 2003-2005 down to as little as 1% in recent years. In terms of World Bank support, Ghana is currently an IDA-blend country [3]. IDA funding is reserved for the world's poorest countries. However, as countries become richer and their creditworthiness improves, they gradually become ineligible for IDA support. Countries that "graduate" from IDA become eligible for support from the International Bank for Reconstruction and Development (IBRD). IBRD funding differs from IDA funding in that it is less concessional; in other words, its lending is closer to market rates. An IDA-blend designation means that a country is in between being fully eligible for IDA support and IBRD funding. This phase of support allows a country to gradually shift from more to less concessional lending terms. However, the time period for remaining a blend country is variable; at the time of writing, there is no clear date for when Ghana will become fully ineligible for IDA funding [4].

The Global Fund has clear transition guidance; however, Ghana does not yet meet the income and disease burden thresholds to initiate transition [5].

In general, many bilateral donors, despite making up most ODA in Ghana, do not have as clear a pathway as multilaterals do to support countries in transitioning out of aid [6, 7]. However, the UK's Department for International Development has made a concerted effort to transition its support away from service delivery and social sectors towards broader governance issues. However, no plan has been made as to when DFID will fully exit from the country [8,9]. Additionally, the Netherlands transitioned its health support several years ago [**Error! Bookmark not defined.**]. In the early 2000s, the Netherlands contributed almost 40% of Ghana's health aid. In 2018, its share was only 1% of total health aid [1].

Appendix II List of interviewees

KI #	Role of the KI
1	Civil society organization
2	Government institution (National)
3	Government institution (National)
4	Government institution (National)
5	Government institution (National)
6	Government institution (National)
7	Government institution (National)
8	Government institution (National)
9	Government institution (National)
10	Civil society organization
11	Donor agency
12	Civil society organization
13	Government institution (Regional)
14	Civil society organization
15	Civil society organization
16	Government institution (Regional)
17	Civil society organization
18	Civil society organization

Notes:

1. Government institutions include Parliament, Ministry of Financing, Ministry of Health and Ministry of Planning (interviewees should hold health related position in these institutions) .

2. The regional government institutions were selected from Accra.

3. Civil society organizations include HIV, Malaria, EPI, and TB related organizations.

Appendix III Interview guide

1. What do you understand donor transitions in Ghana to mean?
2. How would you define a successful transition from external aid within the health sector in Ghana?
   Prompt: ask for any national goals, organizational goals, or personal definitions of what success would look like
3. What is the current state of transition in the health sector in Ghana?
   Prompt: Did (Do) you think Ghana was (is) adequately prepared for transition?
   Follow-up: ask them to give some reasons for their answer
4. How do you feel about Ghana's past/future graduation from external aid?
   Prompt: ask if they feel it's good or bad, and why
5. Based on your experience at your organization, what challenges would [insert name of organization] face as a result of donor transitions in Ghana?
   Prompt: ask for challenges in the following areas – policy, health financing, service delivery, health information and monitoring & evaluation, technical capacity, logistics & supply chain (e.g., vaccines, medicines, technology), human resources (training capacity), governance. Ask for details on how they think these challenges did (will) impact health (access to services, outcomes, etc.)
6. What opportunities for improvement would [insert name of organization] have as a result of donor transitions in Ghana? **NOTE: the focus is on the organization for all respondents. For donors, also ask about organizational opportunities related to transitions in other countries**
   Prompt: ask for opportunities in the following areas – policy, health financing, service delivery, health information and monitoring & evaluation, technical capacity, logistics & supply chain, etc.
   Ask for details on how they think these challenges did (will) impact health (access to services, outcomes, etc.)
7. What lessons, if any, has [organization] learned from its experiences with donor transitions? **NOTE: the focus is on the organization for all respondents**
8. In your opinion, what do you think are the biggest challenges faced by the health sector in Ghana when it experiences donor transitions? How might these challenges impact health (access, delivery, outcomes), especially for vulnerable populations? **NOTE: the focus is on the country for all respondents. For donors, in addition, ask about experiences in other countries**
9. In your opinion, what are the biggest opportunities for the health sector in Ghana when it experiences donor transitions? How might this impact health (access, delivery, outcomes), especially for vulnerable populations? **NOTE: the focus is on the country for all respondents. For donors, in addition, ask about experiences in other countries**
10. In your opinion, do you think there was (is) an opportunity for improved health system efficiency as a result of donor transitions in the health sector?
    If YES, which areas of the health system had (will have) the greatest impact through improved efficiency?
11. In your opinion, what are the biggest obstacles to improving health system efficiency during donor transitions?
    Prompt: political, resource allocation, technical capacity, etc.
12. Can you share any important documents with us?
13. Can you recommend other key informants we can interview about donor transitions in Ghana?

Reference

1 Data analysis by author based on OECD Creditor Reporting System (CRS), Available from: https://stats.oecd.org/Index.aspx?DataSetCode=crs1[Cited on Aug 20, 2020]
2 Gavi, The Vaccine Alliance. Co-financing information sheet: Ghana. 2018 Dec. Available from: https://www.gavi.org/sites/default/files/document/co-financing-information-sheet-ghanapdf.pdf [Cited on Aug 20, 2020]
3International Development Association, World Bank Group. Borrowing countries. Available from: https://ida.worldbank.org/about/borrowing-countries[Cited on Aug 20, 2020]
4 The Center for Policy Impact in Global Health. Health Aid in Transition: A Review of the World Bank International Development Association (IDA). November 2019. Available from: http://centerforpolicyimpact.org/wp-content/uploads/sites/18/2019/11/IDA-Transition-Profile-Final-1.pdf[Cited on Aug 20, 2020]
5 The Global Fund. Projected transitions from Global Fund country allocations by 2028: projections by component. January 2020. Available from: https://www.theglobalfund.org/media/9017/core_projectedtransitionsby2028_list_en.pdf[Cited on Aug 20, 2020]
6The Center for Policy Impact in Global Health. Transitioning away from donor funding for health: a cross-cutting examination of donor approaches to transition. April 2020. Available from: http://centerforpolicyimpact.org/wp-content/uploads/sites/18/2020/04/Cross-cutting-donor-transition-report.pdf[Cited on Aug 20, 2020]
7 ODI. Exit from aid: an analysis of country experiences. April 2019. Available from: https://www.odi.org/publications/11298-exit-aid-analysis-country-experiences[Cited on Aug 20, 2020]
8 Independent Commission for Aid Impact. The changing nature of UK aid in Ghana. Feb 2020. Available from: https://icai.independent.gov.uk/report/ghana/[Cited on Aug 20, 2020]
9 The Center for Policy Impact in Global Health. Health Aid in Transition: A Review of the DFID. July 2020. Available from: http://centerforpolicyimpact.org/wp-content/uploads/sites/18/2020/07/DFID_Aid-Transition-Profile-July-2020.pdf[Cited on Aug 20, 2020]
